# Sorafenib treatment in children with relapsed and refractory neuroblastoma: an experience of four cases

**DOI:** 10.1002/cam4.784

**Published:** 2016-06-05

**Authors:** Keiko Okada, Yoshiko Nakano, Kai Yamasaki, Chika Nitani, Hiroyuki Fujisaki, Junichi Hara

**Affiliations:** ^1^ Department of Pediatric Hematology/OncologyOsaka City General HospitalOsakaJapan

**Keywords:** Metastatic neuroblastoma, pediatric, sorafenib

## Abstract

Metastatic neuroblastoma is an aggressive malignancy with a poor prognosis. Recent findings have shown that sorafenib decreases cell viability and increases apoptosis in human neuroblastoma cell lines. We report an experience of compassionate use of sorafenib in children with treatment‐refractory neuroblastoma. Sorafenib showed transient anti‐tumor activity in all four patients without adverse effects. However, progression was observed after a short stabilization phase. While sorafenib showed minimal anti‐tumor activity in our patients, it might still be effective in patients with neuroblastoma in an earlier stage.

## Introduction

Children with high‐risk neuroblastoma have poor long‐term survival despite intensive multimodal treatment 1, 2. Many early phase clinical trials have been conducted on small‐molecule inhibitors and antibodies, some of which, including anti‐GD2 antibody and ALK inhibitors, may improve outcome 3. Sorafenib is an orally active inhibitor of multiple kinases, including C‐RAF, B‐RAF, c‐KIT, FLT3, platelet‐derived growth factor receptor (PDGFR)‐*α* and ‐*β*, and vascular endothelial growth factor receptor (VEGFR)‐1, ‐2, and ‐3 4. Recent findings have shown that sorafenib decreases cell viability and increases apoptosis in human neuroblastoma cell lines in association with downregulation of ERK, AKT, RAF‐MEK, and JAK2‐STAT3 survival pathways 5, 6. Studies have demonstrated that sorafenib inhibits growth of human neuroblastoma tumor xenografts in mice by targeting both neuroblastoma cells and tumor blood vessels 6, 7. Sorafenib has been evaluated in pediatric patients with solid tumors or leukemia in two phase I studies in combination with other agents 8, 9 and, thereafter, in a Children's Oncology Group phase I trial as single‐agent therapy 10. Hypertension, diarrhea, rash, fatigue, and increased serum levels of alanine aminotransferase (ALT)/aspartate aminotransferase (AST) were the most common sorafenib‐related toxicities. These toxicities and the pharmacokinetic profile were similar to adult studies. The recommended phase II dose of sorafenib was 200 and 150 mg/m^2^ every 12 h for children with solid tumors and leukemia, respectively.

Although the efficacy of sorafenib in children with neuroblastoma is unclear, we considered that compassionate use of sorafenib might be of value in end‐stage neuroblastoma patients with acquired resistance to conventional chemotherapy. We therefore administered sorafenib as compassionate use to children with treatment‐refractory neuroblastoma. Patient eligibility criteria and stopping rules were prepared to prevent overuse and severe adverse events.

## Patients and Methods

Patients <25 years of age with recurrent treatment‐refractory neuroblastoma with either presence of a measurable tumor or a neuron‐specific enolase (NSE) level >20 ng/mL (the upper limit of normal in our institution) were eligible. Other eligibility criteria included Karnofsky/Lansky performance score ≥30%, no hepatic failure, no uncontrolled systemic hypertension, no prior thromboembolic event, and no metastasis within the brain parenchyma. Patients were excluded if they had had active other cancer within 5 years, or if they were pregnant or lactating. This trial was approved by Osaka City General Hospital institutional review board. Informed consent was obtained from parents of the children.

Sorafenib was administered orally, twice daily, continuously for at least 14 days. Capsules (200 mg) were dissolved in warm water for administration to children who could not swallow capsules. The starting dose was 150 mg/m^2^. The dose was rounded off to the nearest multiple of 200 mg. Because sorafenib has a half‐life of >24 h 11, administration of a doubled dose once daily was allowed when a 200‐mg capsule was too much when body surface area was small. If the anti‐tumor effect was insufficient after administration for 14 days without adverse events, dose escalation to 250 mg/m^2^ was allowed according to the physician's decision. Treatment was continued in the absence of marked disease progression. Treatment was stopped if any Grade 4 hematologic or Grade 3–4 nonhematologic toxicity remained after 30‐day discontinuation of sorafenib or if the patient no longer wanted to receive the study drug. Toxicity was graded according to CTCAE v4.0.

## Results

From June 2012 to February 2013, four patients (aged 4–5 years) with relapsed neuroblastoma were treated with sorafenib. Table 1 summarizes their disease characteristics, treatment, and outcome.

**Table 1:** Patient characteristics

Pt no.	Age, year	Sex	*MYCN*	Time to 1st relapse, months	Time from 1st relapse (onset) to sorafenib initiation, months	Salvage therapy	Disease sites at sorafenib initiation	Duration of sorafenib administration, days	Clinical outcome
1	5	M	+	21	12 (33)	CPT‐11, Zol, RTx	Bone (multiple), Liver, Spleen, LN	37	DOD at 34 months from onset
2	5	M	+	21	13 (34)	CPT‐11/IFO, TMZ/ETP, VNR/CPA, RTx	Bone (multiple), Liver, Kidney, Lung, LN	56	DOD at 36 months from onset
3	4	F	−	12	8 (20)	CPT‐11, Zol, TBI	Bone (multiple), LN	21	DOD at 34 months from onset
4	4	F	+	17	16 (33)	CPT‐11, Zol, RTx	Bone (multiple), Pleura, Subcutaneous nodule, LN	33	DOD at 39 months from onset

CPA, cyclophosphamide; CPT‐11, irinotecan; DOD, died of disease; ETP, etoposide; IFO, ifosfamide; LN, lymph node; RTx, radiotherapy; TBI, total body irradiation; TMZ, temozolomide; VNR, vinorelbine; Zol, zoledronic acid.

All patients (age 16–34 months at diagnosis) were classified as International Neuroblastoma Staging System Stage 4. They had adrenal primary tumors and distant metastases in bones, bone marrow, and lymph nodes. All tumors had unfavorable histology and three had amplified *MYCN*. Primary treatments were 4–5 courses of chemotherapy, high‐dose chemotherapy consisting of thiotepa and melphalan with autologous peripheral blood stem cell rescue, radiotherapy, and local surgery. Because their response to primary treatment was insufficient, they had further received allogeneic cord blood transplantation with reduced‐intensity conditioning, and six cycles of 13‐*cis*‐retinoic acid after engraftment. However, they relapsed 12–21 months after disease onset. They subsequently received radiotherapy and several courses of chemotherapy including irinotecan. Efficacies of these salvage treatments were transient or absent, and patients met eligibility criteria of compassionate use. Sorafenib administration was started 269–507 days after relapse. Tumor response to sorafenib could be evaluated in all patients by tumor size and symptoms.

In two patients (Patients 1 and 2), tumor sizes were stable for 3‐4 weeks after the initiation of sorafenib. However, tumor enlargement in the liver (Patient 1, 2) and the spleen (Patient 1) were observed at 4 weeks. Dose escalations to 250 mg/m^2^ per dose were not effective and they died of progressive disease at 44 and 57 days after the initiation of sorafenib. Patient 3 was transiently relieved from tumor‐related pain during first week of administration. However, the pain reappeared shortly after. Dose escalation to 250 mg/m^2^ per dose was not effective and administration of sorafenib was discontinued due to progressive disease. She died of disease after ~1 year. In Patient 4, tumor sizes were stable for first 4 weeks. However, a new pleural metastasis lesion appeared at Day 28 and administration of sorafenib was discontinued due to progressive disease. She died of disease after 4 months.

Hypertension, diarrhea, skin rash, and increased AST/ALT were not observed in any patient.

## Discussion

In this report, four patients with relapsed neuroblastoma were treated with sorafenib. This is the first report on sorafenib treatment for patients with neuroblastoma. Sorafenib showed minimal anti‐tumor activity in our patients with refractory tumors. Our patients did not experience any sorafenib‐related toxicity, during short administration period.

Anti‐tumor activity in our patients was lost after a short stabilization phase. Sorafenib is approved for the treatment of advanced hepatocellular carcinoma by the US Food and Drug Administration. Sorafenib can inhibit hepatocellular carcinoma growth by three mechanisms: blocking tumor cell proliferation via the MAPK pathway; inducing apoptosis by reducing survival factors; and decreasing tumor angiogenesis by inactivation of PDGFR‐*β* and VEGFR‐2/3. However, many patients develop acquired resistance to sorafenib by various mechanisms including activation of the PI3K/AKT pathway, epithelial‐mesenchymal transition, and autophagy 12, 13. In neuroblastoma patients treated with sorafenib, the same mechanisms as for hepatocellular carcinoma might be involved in both the anti‐tumor effect and the acquisition of resistance. Sorafenib might activate alternative survival pathway(s) in neuroblastoma cells. The combination of sorafenib with other agents targeting multiple survival pathways may be necessary to induce more sustained anti‐tumor activity.

In summary, we report an experience of compassionate use of sorafenib in children with treatment‐refractory neuroblastoma. While sorafenib showed minimal anti‐tumor activity in our patients, it might still be effective in patients with neuroblastoma in an earlier stage.

## Conflict of Interest

None declared.
